# Mortality and loss to programme before antiretroviral therapy among HIV-infected children eligible for treatment in The Gambia, West Africa

**DOI:** 10.1186/1742-6405-9-28

**Published:** 2012-10-02

**Authors:** Uduak Okomo, Toyin Togun, Francis Oko, Kevin Peterson, Assan Jaye

**Affiliations:** 1 Medical Research Council (UK) Laboratories, Atlantic Road, Fajara, P.O. Box 273, Banjul, The Gambia; 2 Institute of Tropical Medicine, Nationalestraat 155, 2000, Antwerp, Belgium; 3 WAPHIR Network, Universite Cheikh Anta Diop Laboratoire de Bacteriologie Virologie, Hopital A. le Dantec, 30 Avenue Pasteur, Dakar, Senegal

**Keywords:** Paediatrics, HIV, Pre-antiretroviral therapy, Loss to follow-up, Mortality, Retention, Sub-Saharan Africa

## Abstract

**Background:**

HIV infection among children, particularly those under 24 months of age, is often rapidly progressive; as a result guidelines recommend earlier access to combination antiretroviral therapy (cART) for HIV infected children. Losses to follow-up (LTFU) and death in the interval between diagnosis and initiation of ART profoundly limit this strategy. This study explores correlates of LTFU and death prior to ART initiation among children.

**Methods:**

The study is based on 337 HIV-infected children enrolled into care at an urban centre in The Gambia, including those alive and in care when antiretroviral therapy became available and those who enrolled later. Children were followed until they started ART, died, transferred to another facility, or were LTFU. Cox proportional hazards regression models were used to determine the hazard of death or LTFU according to the baseline characteristics of the children.

**Results:**

Overall, 223 children were assessed as eligible for ART based on their clinical and/or immunological status among whom 73 (32.7%) started treatment, 15 (6.7%) requested transfer to another health facility, 105 (47.1%) and 30 (13.5%) were lost to follow-up and died respectively without starting ART. The median survival following eligibility for children who died without starting treatment was 2.8 months (IQR: 0.9 - 5.8) with over half (60%) of all deaths occurring at home. ART-eligible children less than 2 years of age and those in WHO stage 3 or 4 were significantly more likely to be LTFU when compared with their respective comparison groups. The overall pre-treatment mortality rate was 25.7 per 100 child-years of follow-up (95% CI 19.9 - 36.8) and the loss to programme rate was 115.7 per 100 child-years of follow-up (95% CI 98.8 - 137). In the multivariable Cox proportional hazard model, significant independent predictors of loss to programme were being less than 2 years of age and WHO stage 3 or 4. The Adjusted Hazard Ratio (AHR) for loss to programme was 2.06 (95% CI 1.12 – 3.83) for being aged less than 2 years relative to being 5 years of age or older and 1.92 (95% CI 1.05 - 3.53) for being in WHO stage 3 or 4 relative to WHO stage 1 or 2.

**Conclusions:**

Earlier enrolment into HIV care is key to achieving better outcomes for HIV infected children in developing countries. Developing strategies to ensure early diagnosis, elimination of obstacles to prompt initiation of therapy and instituting measures to reduce losses to follow-up, will improve the overall outcomes of HIV-infected children.

## Background

Combination antiretroviral therapy (cART) has significantly improved the prognosis for HIV-infected children in resource-limited settings [1-4]. Eligibility for ART among children in resource-limited settings is based on either clinical and/or immunological criteria to start treatment at World Health Organization (WHO) clinical stage 3 or 4 disease, or at a CD4 T-cell count/percent below the age-appropriate immunological threshold [5]. However, because of the more rapid disease progression and significantly higher risk of mortality in the first two years of life among HIV-infected children in sub-Saharan Africa [6,7], WHO now recommends that all infants and children aged <24 months with confirmed HIV-infection start ART as soon as possible irrespective of clinical stage or immunological threshold [5,7,8]. Unfortunately, the majority of HIV-infected children in sub-Saharan Africa are diagnosed late with advanced clinical disease and immunosuppression, and are usually 5 years of age or older at initiation of therapy [2,4]. This is due to, among other reasons, the fact that health systems in resource limited settings still face considerable challenges in their efforts to scale-up access to early paediatric HIV diagnosis and treatment, particularly among children aged < 18 months in whom a definitive diagnosis requires sophisticated laboratory techniques. Another challenge that treatment programmes face is ensuring that all children who test HIV-positive are successfully linked to and retained in a Paediatric HIV/AIDS care programme such that they can initiate ART as soon as they are eligible [4,9]. Retention of patients in pre-ART care is of paramount importance in ensuring the success of ART programmes. Loss to care has been defined as “discontinuation of active engagement in pre-ART care for any reason, including death” [10]. Loss to follow-up (LTFU) from HIV care programmes in particular represents missed opportunities for the timely initiation of life-saving treatment. A systematic review of adult ART programmes in sub-Saharan Africa reported that retention is as low as 60% after 2 years [11], which is consistent with our observation of one-third of ART-eligible adults who died or were lost to follow-up prior to initiation of treatment [12]. However, there is a paucity of data on mortality and loss to follow-up experiences of ART-eligible HIV-infected children who fail to initiate treatment because this information is not routinely assessed as part of program evaluations [13-15].

In 2008, the prevalence of HIV-1 and HIV-2 in The Gambia was estimated to be 1.6% and 0.4%, respectively [16]. The prevalence of paediatric HIV/AIDS is amongst the lowest in sub-Saharan Africa with less than 1000 children below the age of 15 years known to be living with HIV/AIDS [17]. Approximately one-third of these are receiving life-saving ART though the proportion that are eligible but are yet to initiate ART is unknown. This study aims to investigate, and determine factors associated with, pre-ART loss to follow-up and mortality among ART-eligible HIV-infected children at the Paediatric HIV clinic of the Medical Research Council (MRC) Unit in Fajara, The Gambia – West Africa.

## Results

### Enrolment, follow-up and ART eligibility

**Figure 1:**
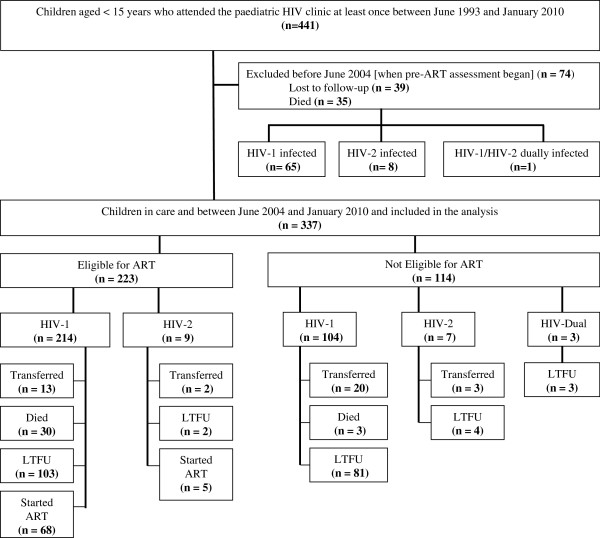
Flow chart of the cohort of HIV-infected children < 15 years of age enrolled in the Paediatric HIV programme, MRC Unit, The Gambia, 1993–2010.

A total of 411 HIV infected children attended the MRC paediatric HIV clinic at least once between June 1993 and January 2010; of these, 74 were excluded from the analysis because they had either died or were LTFU prior to June 2004 when pre-ART sensitization and screening began (Figure 1).

Among the 337 children in care between June 2004 and January 2010 and included in the analysis were 318 (94%) HIV-1 infected, 16 (5%) HIV-2 and 3 (1%) HIV-1/HIV-2 dually infected children. Their median age at diagnosis of HIV infection was 23 months (IQR: 12 – 71 months). Of these children, 126 (37%) were aged less than 18 months, 119 (35.3%) aged between 18 – 59 months and 92 (27.3%) 5 years or older at the time of diagnosis. HIV-2 infected children were older at the time of HIV diagnosis (median 65.6 months; IQR: 26 - 98) than HIV-1 (23.3 months; IQR: 12 – 62) and HIV-1/HIV-2 dually infected children (10.2 months; IQR: 7.6 - 21.8) but this was not statistically significant. 164 (49%) children were female. Overall, 114 (34%) children did not meet ART initiation criteria over this period while 223 (66%) children were found to be eligible for treatment (Figure 1).

### Characteristics of ART-eligible patients

The majority of children in the cohort were enrolled after January 2006 with eligibility based on the 4-stage 2006 revision of the WHO paediatric treatment guidelines (Table 1). 177 (80%) of the children were eligible to start cART at presentation or within 6 months of their being diagnosed with HIV infection; 60% of the children were 2 years of age or older at eligibility. Information on parental vital status was available for 173 of the 223 children, of which half were orphaned: 70 (41%) had lost one parent and 16 (9%) had lost both parents. Only 99 (44%) children had documentation of their parents HIV status - 76 had at least one HIV-positive parent, 17 had both parents HIV-positive and 6 had both parents HIV-negative. Gender, HIV type, CD4% and the vital status of the parents were not significantly associated with the outcomes of survival or retention but there were significant differences by age and WHO clinical status (Table 2); children less than 2 years of age and those in WHO stage 3 or 4 at ART eligibility were significantly more likely to be LTFU when compared with their respective comparison groups.

**Table 1:** Clinical and socio-demographic characteristics of 223 ART-eligible HIV-infected children enrolled in the Paediatric HIV programme, MRC Unit, The Gambia, 2004–2010

**Characteristics**	**n (%)**
**Sex: (n=223)**	
Female	101 (45.3%)
Male	122 (54.7%)
**Eligibility period**^**a**^**: (n= 223)**	
June 2004 – December 2005	42 (18.8%)
January 2006 – January 2010	181 (81.2%)
**Age at eligibility: (n=223)**	
Median in years (IQR)	2.9 (1.4 – 7.4)
< 2 years	89 (40%)
2 – 4 years	49 (22%)
≥ 5 years	85 (38%)
**HIV type: (n=223)**	
HIV-1	214 (95.9%)
HIV-2	9 (4.0%)
HIV-Dual	0
**WHO clinical stage at eligibility: (n=178)**	
1 or 2	63 (35.4%)
3 or 4	115 (64.6%)
**CD4% at eligibility: (n=196)**	
Median (IQR)	11 (7 – 14)
<15%	151 (77.0%)
≥15%	45 (23.0%)
**Haemoglobin at eligibility (n=192)**	
Median (IQR)	9.3 (7.9 – 10.3)
Haemoglobin < 8 g/dl	50 (26%)
**Vital status of parents: (n=173)**	
One parent died	70 (40.5%)
Both died	16 (9.2%)
Both alive	87 (50.3%)

^a^ Defined by age according to the WHO treatment guidelines in effect at the time of eligibility.

**Table 2:** Association of baseline characteristics with outcomes among ART-eligible HIV-infected children enrolled in the Paediatric HIV programme, MRC Unit, The Gambia, 2004–2010

**Variable**	**Started ART**	**Died**	**LTFU**	**Transferred**	**p-value**
**Total (n=223)**	**73 (32.7%)**	**30 (13.5%)**	**105 (47.1%)**	**15 (6.7%)**	
**Sex: (n=223)**					
Female	35 (34.7%)	12 (11.8%)	48 (47.5%)	6 (5.9%)	0.868‡
Male	38 (31.2%)	9 (7.4%)	57 (46.7%)	18 (14.7%)	
**Age at eligibility: (n=222)**					
Median in years (IQR)	4.7 (1.9 – 8.4)	5.1 (1.6 – 10.0)	1.9 (1.2 – 4.7)	7.4 (3.2 – 8.7)	**0.0001**ψ
< 2 years	19 (21.4%)	10 (11.2%)	57 (64.0%)	3 (3.4%)	
2 – 4 years	21 (42.9%)	5 (10.2%)	22 (44.9%)	1 (2.0%)	
≥ 5 years	33 (38.8%)	15 (17.7%)	26 (30.6%)	11 (12.9%)	**<0.001** Ω
**HIV type: (n=223)**					
HIV-1	68 (31.8%)	30 (14%)	103 (48.1%)	13 (6.1%)	0.065 Ω
HIV-2	5 (55.6%)	0	2 (22.2%)	2 (22.2%)	
**WHO stage: (n=178)**					
1 or 2	34 (54%)	8 (12.7%)	16 (25.4%)	5 (7.9%)	
3 or 4	33 (28.7%)	16 (13.9%)	59 (51.3%)	7 (6.1%)	**0.003** Ω
**CD4% at eligibility: (n=196)**					
Median (IQR)	11% (8 – 14)	8% (4 – 11)	11% (8 – 15.0)	13% (8 – 14)	**0.039**ψ
<15%	55 (36.4%)	25 (16.6%)	60 (39.7%)	11 (7.3%)	0.574 Ω
≥15%	18 (40.0%)	4 (8.9%)	21 (46.7%)	2 (4.4%)	
**Haemoglobin: (n=192)**					
Median (IQR)	9.6 (7.9 – 10.6)	9.5 (7 – 11.4)	9.1 (8.1 – 9.9)	10 (8.6 – 10.5)	0.262ψ
< 8 g/dl	20 (40%)	9 (18%)	20 (40%)	1 (2%)	0.245 Ω
**Vital status of parent: (n=173)**					
Single orphan	25 (35.7%)	6 (8.6%)	31 (44.3%)	8 (11.4%)	
Double orphan	6 (37.5%)	3 (18.7%)	4 (25%)	3 (18.8%)	
Both alive	37 (42.5%)	9 (10.4%)	39 (44.8%)	2 (2.3%)	0.073 Ω

‡ Chi-square test.

Ω Fishers exact test.

ψ Kruskall-Wallis test.

### Survival and retention in care among ART-eligible patients

Among the 73 children who commenced cART; the median time from eligibility to ART initiation and median age at initiation were 5.1 months (IQR: 2.8 – 11.2) and 4.9 years (IQR: 2.6 – 9.9) respectively.

Thirty (13.3%) ART-eligible children died without staring treatment giving a pre-treatment mortality rate of 25.7 per 100 child-years of follow-up (95% CI 19.9 – 36.8). The median survival following ART eligibility for children who died without starting treatment was 2.8 months (IQR: 0.9 – 5.8) with over half (60%) of all deaths occurring at home (Table 3).

**Table 3:** Characteristics of 30 treatment eligible children who died prior to the initiation of ART

**Cause of death**	**Age at eligibility**	**Sex**	**WHO clinical stage at eligibility**	**CD4% or absolute CD4 count at eligibility**	**Place of death**
Disseminated Tuberculosis, Sepsis	13 years	M	1	140 cells/mm^3^	Hospital
Disseminated Tuberculosis, Sepsis	8 years	M	3	10 cells/mm^3^	Hospital
Kaposi Sarcoma	7 years	M	4	-	Hospital
Severe Malnutrition	8 months	M	4	22%	Hospital
Severe Malnutrition, Sepsis	19 months	M	4	27%	Hospital
Severe Malnutrition, Sepsis	8 years	F	4	120 cells/mm^3^	Hospital
Tuberculosis	19 months	F	3	13%	Hospital
Tuberculosis	2 years	M	3	9%	Hospital
Tuberculosis	7 months	M	3	10%	Hospital
Tuberculosis	6 years	M	3	30 cells/mm^3^	Hospital
Tuberculosis, Meningitis	11 years	M	4	10 cells/mm^3^	Hospital
Very Severe Pneumonia	8 months	M	3	8%	Hospital
Unknown	2 years	F	2	3%	Home
Unknown	4 years	F	-	3%	Home
Unknown	20 months	M	4	9%	Home
Unknown	1 month	F	1	10%	Home
Unknown	13 months	F	3	19%	Home
Unknown	23 months	F	-	7%	Home
Unknown	2 years	F	4	16%	Home
Unknown	3 years	M	2	13%	Home
Unknown	19 months	F	-	8%	Home
Unknown	13 years	M	-	20 cells/mm^3^	Home
Unknown	14 years	M	2	320 cells/mm^3^ (8%)	Home
Unknown	10 years	F	1	20 cells/mm^3^	Home
Unknown	6 years	M	1	160 cells/mm^3^	Home
Unknown	10 years	M	1	10 cells/mm^3^	Home
Unknown	12 years	M	-	380 cells/mm^3^ (11%)	Home
Unknown	12 years	F	3	60 cells/mm^3^	Home
Unknown	6 years	M	4	540 cells/mm^3^	Home
Unknown	9 years	F	-	90 cells/mm^3^	Home

One hundred and five (47%) treatment-eligible children who remained in the cohort for a median of 4.2 months (IQR: 3.4 – 5.3) were eventually LTFU before starting treatment. Nineteen (18%) of these children were last seen on the date that that they were diagnosed HIV-positive and/or assessed to be eligible for ART and of these, 14 were less than 2 years of age. WHO clinical stage at eligibility was documented in 75 of those lost to follow-up and 25 (33%) and 34 (45.3%) had a stage 3 and stage 4 condition respectively. Twenty-two (21%) of the children LTFU had one or both parents in HIV care whilst 35 (33%) were either single or double orphans.

**Figure 2:**
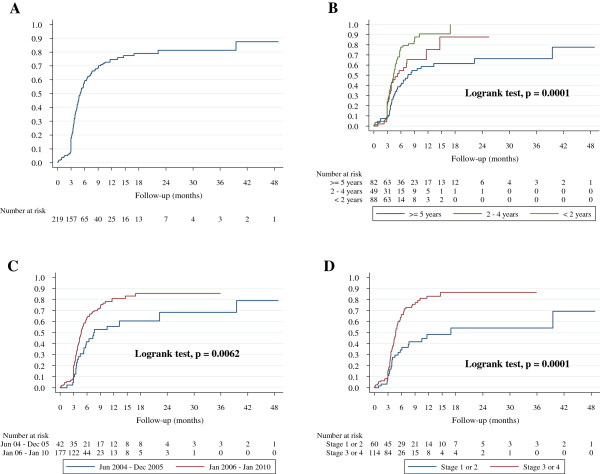
**Cumulative incidence of loss to programme following eligibility for cART among HIV-infected children enrolled in the Paediatric HIV programme, MRC Unit, The Gambia, 2004 – 2010.** Plot **A** shows the cumulative incidence of loss to programme before starting treatment for all ART-eligible patients; Plots **B**, **C**, and **D** show significant unadjusted associations between age category, WHO clinical stage and eligibility period respectively, and the risk of becoming lost to the programme.

A total of 135 ART-eligible children were lost to the programme before ART initiation giving an incidence rate for the composite endpoint of death or LTFU of 115.7 per 100 child-years of follow-up (95% CI 98.8 – 137). In both univariate and multivariable analyses of risk factors for death, no significant risk factors were identified (Table 4); however being less than 2 years of age at eligibility, having advanced clinical HIV disease (i.e. WHO stage 3 or 4) and having been assessed as eligible for ART between January 2006 and January 2010 were all significantly associated with loss to programme. Figure 2 shows the cumulative incidence of loss to programme overall, by age category, eligibility period and WHO clinical stage at ART eligibility. The graphs show significant unadjusted associations between age category (log rank test, p = 0.0001), eligibility period (log rank test, p = 0.0062), WHO clinical stage (log rank test, p = 0.0001) and loss to programme risk from the time of being assessed as ART-eligible. The unadjusted Hazard Ratio for loss to programme was 2.41 (95% CI 1.61 – 3.61) for children less than 2 years of age at the time of eligibility compared with children 5 years of age or older; 2.43 (95% CI 1.51 – 3.90) for being in WHO stage 3 or 4 relative to WHO stage 1 or 2 and 1.86 (95% CI 1.18 – 2.93) for being assessed as eligible for ART between January 2006 and January 2010 compared to the period between June 2004 and December 2005 (Table 4). In the multivariable Cox proportional hazard model, significant independent predictors of loss to programme were being less than 2 years of age and WHO stage 3 or 4. Being less than 2 years of age is the strongest independent predictor of loss to programme among ART-eligible patients - the Adjusted Hazard Ratio (AHR) for loss to programme was 2.06 (95% CI 1.12 – 3.83) for being aged less than 2 years relative to being 5 years of age or older and 1.92 (95% CI 1.05 – 3.53) for being in WHO stage 3 or 4 relative to WHO stage 1 or 2. The Proportional hazards assumption was met for all Cox proportional hazard models.

**Table 4:** Risk factors associated with death and loss to programme before initiation of ART among HIV-infected children eligible for ART in the Paediatric HIV programme, MRC Unit, The Gambia, 2004–2010

**Patient Characteristics**^**b**^		**Death**				**Loss to programme**			
		**Crude HR 95% (CI)**	***P***	**Adjusted HR 95% (CI)**	***P***	**Crude HR 95% (CI)**	***P***	**Adjusted HR 95% (CI)**	***P***
**Sex: (n=219)**									
Female	99 (45.2%)	1.0		1.0		1.0		1.0	
Male	120 (54.8%)	1.16 (0.56 – 2.41)	0.686	1.23 (0.43 – 3.51)	0.700	0.94 (0.67 – 1.32)	0.731	1.15 (0.68 – 1.95)	0.600
**Age category: (n=219)**									
< 2 years	88 (40.2%)	0.86 (0.38 – 1.94)	0.712	1.64 (0.50 – 5.38)	0.415	2.41 (1.61 – 3.61)	**<0.0001**	2.06 (1.12 – 3.83)	**0.023**
2 – 4 years	49 (22.4%)	0.66 (0.24 – 1.83)	0.429	0.50 (0.09 – 2.64)	0.410	1.58 (0.97 – 2.58)	0.069	0.95 (0.43 – 2.10)	0.896
≥ 5 years	82 (37.4%)	1.0		1.0		1.0		1.0	
**WHO clinical stage: (n=174)**									
1 or 2	60 (34.5%)	1.0		1.0		1.0		1.0	
3 or 4	114 (65.5%)	1.49 (0.63 – 3.55)	0.367	2.98 (0.78 – 11.31)	0.109	2.43 (1.51 – 3.90)	**<0.0001**	1.92 (1.05 – 3.53)	**0.035**
**CD4%: (n=193)**									
<15%	148 (76.7%)	1.69 (0.59 – 4.86)	0.330	1.26 (0.39 – 4.09)	0.700	0.90 (0.58 – 1.42)	0.662	1.23 (0.67 – 2.26)	0.499
≥15%	45 (23.3%)	1.0		1.0		1.0		1.0	
**Vital status of parent: (n=169)**									
Single orphan	68 (40.2%)	0.75 (026 – 2.11)	0.582	0.71 (0.23 – 2.20)	0.548	0.89 (0.58 – 1.38)	0.612	0.90 (0.52 – 1.57)	0.720
Double orphan	16 (9.5%)	1.93 (0.52 – 7.17)	0.323	1.32 (0.30 – 5.82)	0.711	0.95 (0.43 – 2.10)	0.903	1.12 (0.46 – 2.70)	0.800
Both alive	85 (50.3%)	1.0		1.0		1.0		1.0	
**Eligibility period: (n= 219)**									
Jun 2004 – Dec 2005	42 (19.2%)	1.0		1.0		1.0		1.0	
Jan 2006 – Jan 2010	177 (80.8%)	1.19 (0.50 – 2.83)	0.690	3.59 (0.40 – 32.4)	0.254	1.86 (1.18 – 2.93)	**0.007**	2.06 (0.86 – 4.96)	0.106

^a^ Children were lost to the programme if they died or were lost to follow-up.

^b^ 4 children excluded from the analysis: 1child had an of incorrect date of HIV eligibility while 3 children had incorrect date of exit.

Proportional hazards assumption was met for all Cox proportional hazard models as assessed by the global test based on the Schoenfeld residuals.

Ф Adjusted for Sex, Age, WHO stage, CD4% and eligibility period.

### Outcomes among HIV-infected children ineligible for ART at enrollment

The 114 children who did not meet ART initiation criteria over the study period were followed up for a median of 1.7 months (IQR: 0.2 – 16.9) from enrolment. Twenty-three (20.2%) children were transferred to another clinic, 88 (77.2%) were LTFU and 3 (2.6%) died; all deaths occurred within three months of enrolment. All these children had been given follow-up appointments no later than 3 months after their last appointment, and were explicitly given permission to return earlier to the clinic in case of illness.

## Discussion

Our study reports pre-treatment mortality and losses to follow-up among ART-eligible children from a cohort who were yet to initiate treatment at a West-African clinic. The majority of deaths in our cohort occurred in the first three months after eligibility and during the period of preparation for ART. The pre-treatment mortality rate observed in our setting is relatively higher than reports from other sites; a study in rural Zambia reported 10% mortality among ART-eligible children with a mortality rate of 2.73 (95% CI: 1.7 – 4.18) per 100 child-years [14] while another study reported that 1% of treatment-eligible children receiving care at an urban ART program in Zambia died prior to ART initiation [18]. In a large cohort of 1766 children in Cote d’Ivoire, the reported loss-to-programme rate of 50.3/100 child-years of follow up was also much lower than that reported in this study though, it was not clear if the children in that cohort were eligible for ART. One possible reason for the relatively higher pre-treatment mortality and loss-to-programme rates among ART-eligible children in our setting is the advanced stage of clinical disease and immunosuppression at diagnosis. Many children in resource limited settings are only tested for HIV as part of workup for recurrent or severe ill health; we reported that 80% of the treatment-eligible children attending our clinic were assessed as eligible for ART either at or within 6 months of their being diagnosed with HIV infection. The inclusion of home visits as part of our patient follow-up protocol provides a more accurate and complete ascertainment of survival status that could have contributed to the higher mortality rates observed in our cohort.

The proportion of children who initiated ART in our clinic is comparable to that reported in a study in South Africa where 39.5% (96/243) of ART-eligible children started treatment [19] but considerably lower than figures reported from Cote d’Ivoire [15] and Zambia [14] where 86% and 70% of ART-eligible children respectively started therapy. The median time of 5.1 months from eligibility to ART initiation in our cohort was comparable to that reported from Cambodia where ART was initiated after a median of 4.7 months [13], but more than twice as long as the 2.1 months reported from rural Zambia [14]. Another study from Zambia reported that children eligible for ART at presentation in rural areas took longer time to initiate treatment than children in urban areas (3.6 vs. 0.9 months) [18].

Delays in treatment initiation among eligible children are reported to be due to several factors [12-14,19,20] some of which were observed in our cohort. Firstly, the long process of pre-treatment counselling required repeated clinic visits to ensure that care-givers are both willing and capable of taking the responsibility of supporting the child to take life-long medications with a high level of adherence. Lack of caregiver readiness as assessed during counselling sessions could prolong the process further. This often proved difficult for patients from rural areas and those with working parents though parents/caregivers who were in HIV care, specifically those already ART and with good adherence, underwent ‘accelerated’ counselling with emphasis on the challenges of administering drugs to children, drug-storage and side-effects. We however did not collect information on the number of parents/caregivers who completed the 4 pre-ART counselling sessions among children who died or were LTFU.

A second factor for treatment delay was the time required to obtain approval to start ART from the national ART eligibility committee. The fortnightly occurrence of the meeting frequently resulted in eligible children, whose caregivers had completed pre-ART counselling, waiting for days (sometimes as long as one month if the meeting date fell on a public holiday) to receive approval to start treatment. The concept of the national eligibility committee arose in the early days of ART availability in The Gambia when ART supplies were limited, providers inexperienced and practical application of eligibility criteria was still being debated. Though the committee now meets weekly, the inconvenience of travel to attend the meeting, increased uptake of HCT services nationwide and the widespread availability of ART favour decentralization of the committee to centres involved in provision of ART and may help shorten the time patients have to wait in order to initiate life-saving treatment.

Thirdly, caregivers failed to attend follow-up appointments; almost 20% of treatment eligible children attended only one clinic visit before being lost to follow-up. Poor uptake of paediatric HIV services is multifactorial and reasons include facility-related factors such as long queues, overcrowding, negative staff attitudes; unemployment, lack of money for food and transport, difficulty getting time off work, absenteeism from school and fear of social rejection from disclosure to family and neighbours [21]. While the cost of transport was not a hindrance to clinic attendance in our setting due to the practice of reimbursement of transport costs for all patients, disclosure of HIV-status to a family member or friend was a major factor for loss to follow-up among our adult cohort [12] and suggests that unwillingness to disclose the child’s status to another caregiver may have contributed significantly to failure to keep clinic appointments as well as delays in initiation of ART among eligible children. Disclosure of paediatric infection is multifaceted being dependent on caregiver and family characteristics such asbiologic relationship, caregiver permanence, caregiver beliefs and psychosocial function, as well as child-specific factors such as age, developmental stage, cognitive abilities and psychosocial function [22]. A child’s positive status usually indicates maternal infection and the resultant anxiety, fear of blame, social and healthcare discrimination as well as marital abandonment may negatively influence the mother’s acceptance of the HIV status of the child [21,23]. Denial of results, despair or depression hinder health-care seeking and social support, and may lead to difficulties in reacting to the options and advice given by health workers [23-25]. Maternal depression (in cases of vertical transmission) and concurrent HIV or other comorbidities may also affect the mother’s ability to care for the child; the belief that the child might die any moment may cause her not to take proper care of the child anymore. In circumstances where the biologic parents may have died or may be too ill to care for the child, responsibility for care is shifted to one or more relatives or family friends; poor coordination amongst multiple caregivers may also compromise the quality of care especially where secondary caregivers have not been disclosed to and therefore do not understand the importance of regular clinic visits or adherence to prescribed medications [21]. Studies from sub-Saharan Africa suggest that whilst many caregivers appreciate shared childcare, sustained adherence and community support as benefits of disclosing a child’s HIV status to others, many still believe that disclosure to others could have negative effects [21,23,26]. Community-based support groups have been shown to play a major role in providing continuous support to caregivers of HIV-infected children [27] and though several support groups were linked to our clinic, we did not routinely collect data of patient/caregiver membership with such groups or assess what impact this had on their quality of life. Disclosing a diagnosis of HIV infection to a child has been suggested to have direct benefits for adherence to ART and among adolescents is associated with higher retention in care [26,28,29]. The average age of disclosure reported among children in sub-Saharan Africa is 8 years [30,31]; 75% of those LTFU in our cohort (data not shown) were less than 5 years of age at eligibility and therefore too young for a disclosure their HIV status.

Lastly, we report that tuberculosis was the most common cause of death among treatment-eligible children and may have also been an underlying cause of death among those who reportedly died at home. This is consistent with data from paediatric cohorts in resource limited settings in which tuberculosis has been cited as a major reason for delayed treatment initiation [19,20] as well as the most common co-morbidity associated with early deaths among both ART naïve and experienced children [13,14,32]. As such, treatment for concurrent tuberculosis could also have contributed to the delay in initiation of ART.

Overall, 60% of ART-eligible children in our programme died or were lost to follow-up without starting treatment giving a loss-to-care incidence rate of 128 per 100 child-years of follow-up. We observed that the children who were lost to care were significantly more likely to be less than 2 years of age at ART eligibility or to have advanced clinical disease (predominantly tuberculosis and severe malnutrition - data not shown). Children aged less than 2 years or in WHO stage 3 or 4 had almost twice the risk of being lost to the programme before starting treatment respectively compared with those 5 years of age or older and those in WHO stage 1 or 2. The observation that just over half of children LTFU were in WHO stage 3 or 4 coupled with the relatively shorter median time to LTFU from eligibility compared with the time to initiation of ART (4.2 vs 5.1 months), suggests that many of those LTFU may have died as a result of rapid disease progression and advanced HIV disease during the time of preparation for ART and may have not even completed the process. This observation supports the 2010 WHO recommendation that ART be initiated for all HIV-infected infants and children between 12 and 24 months of age irrespective of their immunological threshold or clinical stage [5]. In our study the vital status of the parent was not related to death or loss to care, which was surprising as HIV-infected orphans in sub-Saharan have been shown to have delayed access to HIV care and treatment as well as reduced clinic attendance [33,34] .

Children assessed as eligible for ART between January 2006 and January 2010 using the revised 2006 guidelines were observed to have had a significantly higher risk of being lost to programme compared with those assessed as eligible between June 2004 and December 2005 based on the 2003 WHO treatment guidelines. As the paediatric cohort was a sub-cohort of a much larger adult cohort, this may be attributable to the increase in clinic enrolment over time and the reduced ability of clinic staff to effectively manage the patient load. This risk remained albeit insignificantly after adjusting for the effect of age, clinical stage and other variables.

As the only centre in the country performing virological tests for the diagnosis of HIV infection in children <18 months of age and the major paediatric HIV referral clinic in The Gambia, we experienced a good uptake of paediatric HIV services at our HIV clinic, which however, is not representative of paediatric HIV care in West Africa as patients had the advantage of reimbursement of all transport costs incurred in clinic visits, free treatment, nutritional support and provision and school fees for all children enrolled in school up till the age of 18 years. This coupled with the small size of our paediatric cohort, limit the generalizability of findings. There were however several limitations to this study; data were incomplete for many key variables such as WHO stage or CD4 T-cell percentage at eligibility and these children were classified based on immunologic or clinical criteria alone. Secondly, children with moderate or severe malnutrition in our setting were initially classified as WHO stage 3 or 4 respectively without assessing their response to nutritional support and treatment and thus may have been misclassified as eligible for treatment. Unreported deaths among ART-eligible children LTFU in our cohort suggest that the reported mortality rate may be underestimated; in addition, a good proportion of the children ineligible for ART and also LTFU may have progressed to an advanced stage of HIV-disease and died as they became eligible.

## Conclusion

The results of our study have shown that HIV-infected children in The Gambia are enrolled into care at an advanced stage of disease with severe immunosuppression and a significant number of these treatment-eligible children die before initiating therapy. Only one-third of ART-eligible children go on to initiate ART but face a number of out-of-program and in-program delays before treatment is eventually commenced. Developing strategies to ensure early identification of HIV-infected children before they become eligible for ART, elimination of obstacles to prompt initiation of therapy as well as instituting measures to reduce losses to follow-up, will improve the overall outcomes of HIV-infected children.

## Methods

### Study setting and participants

This study was conducted in MRC HIV research clinic located in the urban area of Greater Banjul, the Gambia. The clinic established an adult cohort in 1986 with approval from the Joint Gambian Government/MRC Ethics Committee and started enrolment of children in 1993 based on written informed consent by the parent or legal guardian, with the adoption of a family-centred model of HIV-care. Paediatric patients consist of exposed or infected infants and children of adults enrolled in the MRC cohort, children with a positive HIV serologic test referred to the clinic from the MRC Clinical services or other health-care services in the country. HIV-exposed infants referred from other PMTCT programmes were also enrolled in the paediatric clinic. Provision of ART in The Gambia began in October 2004 through the support of the Global Fund. Nutritional support is provided and school fees for all children enrolled in school up till the age of 18 years are paid. All treatments, including cART, and treatment monitoring are provided free of charge and the parents/guardians of enrolled children also receive reimbursement of transport costs incurred in clinic visits.

Acquisition of clinical data in an electronic system includes capture of demographic characteristics, social history, basic anthropometric measurements, complete blood count, serum biochemistry, CD4 T-cell profile, chest radiograph, opportunistic/concomitant infections and clinical stage. Patients are required to be seen in clinic at least once every 3 months for follow-up clinical assessment, and CD4 T-cell monitoring is performed every 6 months or earlier if clinically indicated. All children receive daily cotrimoxazole prophylaxis [35,36].

### Selection for ART

Eligibility for ART was determined using immunological and/or clinical criteria based on the Gambian National ART guideline [37] and WHO paediatric ART guidelines that were in place at the time of enrolment; for children enrolled on or before December 31 2005, eligibility was based on the 3-stage 2003 guidelines while for those enrolled from January 2006 eligibility was based on the 4-stage 2006 revision of the guidelines [38,39]. Following a further revision of the treatment guidelines in June 2008 [WHO 2008], ART was recommended for all children <12 months of age with confirmed HIV infection. In addition, all children were required to have at least one identifiable caregiver who would serve as a ‘treatment supporter’ and take responsibility for the administration of the child’s medications. Pre-ART counselling was conducted twice a week and caregivers underwent a minimum of four one-on-one counselling sessions over a period of three to six weeks before selection was processed and confirmed by the national eligibility committee.

Patients who did not come to the clinic for at least 90 days beyond their last scheduled visit were considered LTFU and visited at home by trained field workers to ascertain survival status or change of address. For children who died at home, deaths were verified by close family members and cause of death ascertained by verbal autopsy. Recruitment of children into the programme ended in January 2010 as part of the transition process to transfer patient care from the MRC to the Gambian national health care system.

### Laboratory methods

#### HIV diagnosis and CD4 measurement

Screening for HIV-1 and HIV-2 infection was done using a protocol described in detail elsewhere [40]. In children <18 months of age, HIV infection was diagnosed by two polymerase chain reaction (PCR) tests. Percentages and absolute counts of CD4 T lymphocytes were determined on a FACS Calibur (Becton Dickinson, US) using BD MultiTest reagents and MultiSet software (BD Immunocytometry Systems).

### Statistical analysis

For the purpose of this analysis, eligibility for ART was determined from June 2004 when sensitization of patients began in preparation for the roll-out of ART, to January 2010 when patient recruitment ended. Children already in the paediatric cohort and eligible for treatment but who died or were lost to follow-up before June 2004 were excluded from the analyses. Severe anaemia was defined as haemoglobin <8 g/dl [41]. Distributions of categorical variables were compared between the four possible outcomes: [a] initiation of ART; [b] death; [c] loss to follow-up or [d] transfer by chi square statistics or Fisher exact test as appropriate. Continuous variables were compared using Kruskall-Wallis non-parametric test.

Follow-up duration in person-time was calculated from the date the children were assessed as eligible for ART; children who started ART were right-censored on the date treatment started; those who requested transfer were followed through to the date of transfer. Patients who died were followed to their date of death if known or date last seen alive. To avoid immortal person-time bias, patients LTFU were right-censored 90 days after the date of their last clinic visit (as loss to follow-up was not possible before this time). The probability of loss to programme following ART-eligibility was described using cumulative incidence curves and summarised by mortality rate and loss to programme rate (incident rate for the composite endpoint of death and LTFU) reported per 100 child-years of follow-up respectively. Cox proportional hazards regression models were used to identify baseline characteristics associated with death or a loss to programme. The proportional hazards assumption was assessed for all models by the global test based on the Schoenfeld residuals. All statistical analyses were performed with STATA release 11.1 software (Stata Corp., College Station, TX, USA) and statistical significance defined as p < 0.05 (two-sided).

## Abbreviations

AHR: Adjusted Hazard Ratio; AIDS: Acquired Immune Deficiency Syndrome; cART: Combination Antiretroviral Therapy; CHR: Crude Hazard Ratio; CI: Confidence Interval; HCT: HIV Counselling and Testing; HIV: Human Immunodeficiency virus; IQR: Inter-quartile Range; LTFU: Loss to follow up; MRC: Medical Research Council; PCR: Polymerase chain reaction; PMTCT: Prevention of mother-to-child transmission; WHO: World Health Organization.

## Competing interests

The authors declare that they have no competing interests.

## Authors’ contributions

UO coordinated the pediatric HIV clinical activities, performed the statistical analysis and drafted the manuscript. TT contributed to the study design, participated in the statistical analysis and helped to draft the manuscript. TT, FO and KP participated in the clinical care of the patients. AJ conceived the idea, edited the manuscript and gave overall support. All authors read and approved the final manuscript.
